# Application Value of Spectral CT Imaging in Quantitative Analysis of Early Lung Adenocarcinoma

**DOI:** 10.1155/2022/2944473

**Published:** 2022-03-16

**Authors:** Wang Du, Mingji Yu, Xiaojie Luo, Min Chen

**Affiliations:** ^1^Department of Radiology, Beijing Hospital, National Center of Gerontology, Institute of Geriatric Medicine, Chinese Academy of Medical Science, Beijing 100730, China; ^2^Department of Radiology, Jiangwan Hospital, Shanghai, China

## Abstract

**Objective:**

To investigate the clinical value of gemstone energy spectral CT imaging for the quantitative analysis of early lung adenocarcinoma.

**Methods:**

76 cases of pulmonary ground-glass nodules pathologically confirmed as precancerous lesion and early lung cancer (including pure ground-glass nodules in 46 cases and mixed ground-glass nodules in 30 cases) underwent chest CT scan first and then underwent contrast-enhanced gemstone energy spectral CT to get arterial phase images, venous phase images, and delayed phase images. All the lesions were set the region of interest (ROI). Cases of the pure ground-glass nodule (pGGN) were measured at the maximum level of lesions, cases of the mixed ground-glass nodule (mGGN) were measured in two areas of ground-glass and solid components, CT value and iodine concentrations of lesions in three-phase scanning were separately measured, and at the same time, iodine concentrations of the thoracic aorta were also measured. The normalized iodine concentrations (NICs) were calculated, that is, the ratio of iodine concentrations of lesions and the thoracic aorta. CT values of lesions were also measured at each stage of 70 keV. All the quantitative data were expressed by the mean ± standard deviation, and paired *t*-test was used for pairwise comparison.

**Results:**

In 76 cases, in the spectral CT imaging mode, the NIC value of solid components of the GGN was 0.33 ± 0.16 in the arterial phase (AP), 0.52 ± 0.25 in the venous phase (VP), and 0.58 ± 0.34 in the delayed phase (DP). There were significant differences of *P* values of NICs between each two phases in both solid component cases and the ground-glass component cases in AP/VP, VP/DP, and AP/DP (*P* < 0.05); there were no statistically significant *P* values of CT values between each two phases in three-period enhanced CT in both the solid component cases and the ground-glass component cases in AP/VP, VP/DP, and AP/DP (*P* > 0.05).

**Conclusion:**

Gemstone energy spectral CT with quantitative imaging can dynamically reflect the enhancement features of the pulmonary GGN.

## 1. Introduction

Pulmonary ground-glass nodule (GGN) is an important manifestation of early lung adenocarcinoma. Studies have shown that a considerable number of pulmonary GGNs are early lung adenocarcinoma or precancerous lesions [1], and the malignant rate of GGNs is higher than that of solid pulmonary nodules (SPNs). The development of high-resolution CT (HRCT) and the popularization of lung cancer screening lead to a higher detection rate of GGNs and more attention to it among people. The correct diagnosis and early treatment of early lung cancer and precancerous lesions are of great significance to improve the survival and quality of life of patients with lung cancer. Study on images of the GGN at home and abroad mainly focuses on the analyses of morphological characteristics of lesions based on the CT plain scan and follow-up according to the growth rate of lesions. However, the enhanced CT scan is mostly focused on the SPN, with limited research on the GGN.

Gemstone spectral imaging (GSI) has been a new imaging technique in recent years, which can reconstruct images of single energy in 40–140 keV and separate the components of tissues. GSI provides an analytical tool and quantitative indexes for early lung adenocarcinoma of GGNs. This study aimed to explore the value of GSI in the enhanced scan of pulmonary GGNs. After enhanced CT scanning with GSI was performed on pulmonary GGNs, the data were quantitatively analyzed to display the lesions and characteristics of the pathological tissues more comprehensively.

## 2. Materials and Methods

### 2.1. General Data

From July 2016 to March 2018, 76 cases (30 males and 46 females; age: 56.4 ± 8, range: 35–72) of pulmonary GGNs were collected, which underwent contrast-enhanced gemstone spectral CT and were verified by operation. They do not have any history of significant pulmonary diseases, including chronic obstructive pulmonary disease, asthma, and pulmonary tuberculosis. Among the 76 cases, 46 were pure GGN (pGGN), and 30 were mixed GGN (mGGN). The sizes of lesions were 0.3–3.0 cm, including 19 cases of atypical adenomatous hyperplasia (AAH), 23 cases of adenocarcinoma in situ (AIS), 12 cases of microimpregnated adenocarcinoma (MIA), and 22 cases of invasive adenocarcinoma (IAC). All cases were pathologically confirmed by surgery, thoracoscopy, or CT-guided percutaneous biopsy.

The independent ethic review board approved the study protocol. All patients had signed their informed consent.

### 2.2. Gemstone Spectral CT Scanning

All patients underwent lung scanning with the HDCT scanner (Discovery 750, General Electric Company, GE, US). First, the chest scanning was performed from the entrance of the chest to the diaphragm of basis pulmonis. Then, gemstone spectral CT three-phase enhanced scanning was performed. The tube voltage was switched instantaneously between 80 and 140 kVp. Nonionic contrast agent (iodophenol, containing 300 mg/ml iodine) was injected through the elbow vein by using a double-syringe power injector, with the dose of 80–100 ml and flow rate of 4 ml/s, and 20 ml of normal saline was injected, with the flow rate of 4 ml/s. Arterial phase, venous phase, and delayed phase were performed 35 s, 75 s, and 120 s after the injection of contrast agents, respectively. The layer thickness and interval were both 5.00 mm, with FOV 400 mm ∗ 400 mm and 1.25 mm of the layer thickness for image reconstruction. Data processing and analysis were performed with GSI Viewer software package in ADW 4.4 workstation, through which single-energy images and iodine-water-based images can be obtained in 40–140 keV.

### 2.3. Image Postprocessing and Data Measurement

GSI monoimages of 1.25 mm reconstructed with the gemstone spectral CT were delivered to the ADW 4.4 workstation, and the GSI Viewer package was started. Region of interest (ROI) was drawn for all lesions on the single-energy image (70 keV, 1.25 mm). Measurement for pGGO cases was performed on the layer of the largest lesion, and two regions of ground-glass and solid components were measured for mGGO cases. The vascular, calcification, and vacuolar areas were avoided in all ROI regions, and ensure that the positions and sizes of ROI regions in the three-phase single-energy images were the same for one lesion.

The iodine concentrations (ICs) of foci in the arterial phase, venous phase, and delayed phase were measured on the iodine-based images of iodine (water)-based substance separation. Then, IC of the descending aorta in the same layer was measured, and the normalized iodine concentrations (NICs) were calculated. NIC = IC of foci/IC of the descending aorta in the same layer.

### 2.4. Statistical Analysis

All quantitative data were presented as the mean ± standard deviation (SD) and processed with SPSS version 19.0 (SPSS Inc., Chicago, IL). Pairwise comparisons were conducted using a paired-sample *t*-test with an *α* = 0.05 two-sided significance level. *P* < 0.05 was considered statistically significant.

Of the 76 GGN cases, 46 were pGGNs and 30 were mGGNs. The CT value and NIC in the GGN group in the arterial phase, venous phase, and delayed phase showed more solid components than ground-glass components (Tables 1 and 2).

In the arterial phase, venous phase, and delayed phase, pairwise comparisons were performed among NIC values of the solid and ground-glass components of the GGN in the spectral mode. *P* values of NIC in solid components of the GGN were 0.010, 0.006, and 0.000 in the arterial/venous phase, venous/delayed phase, and arterial/delayed phase, respectively, with statistically significant differences among all the phases (*P* < 0.05) (Table 3). Also, *P* values of NIC in ground-glass components of the GGN were 0.000, 0.005, and 0.001 in the arterial/venous phase, venous/delayed phase, and arterial/delayed phase, respectively, with statistically significant differences among all the phases (*P* < 0.05) (Table 4).

In the arterial phase, venous phase, and delayed phase, pairwise comparisons were performed among CT values of the solid and ground-glass components of the GGN, suggesting no statistical significance (*P* > 0.05) (Tables 3 and 4).

## 3. Discussion

**Figure 1:**
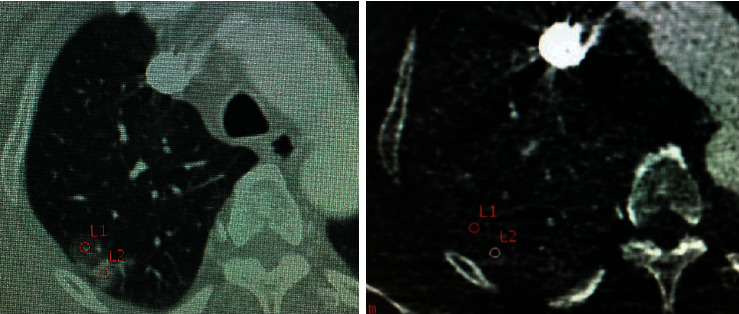
Female, 56 years old, adenocarcinoma in the right upper lobe. Schematic diagram of the ROI. Single-energy and iodine-based images showed the mGGN.

**Figure 2:**
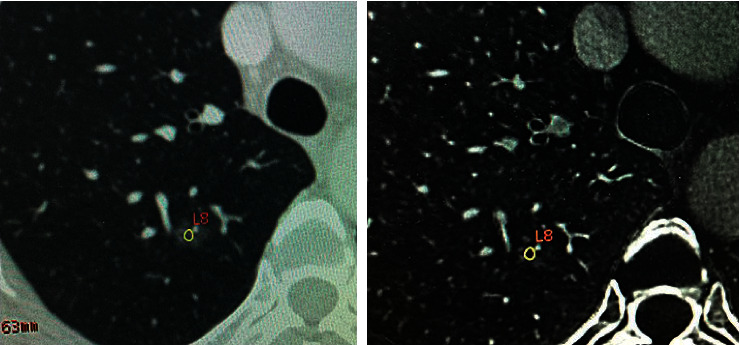
Female, 62 years old, atypical hyperplasia in the right upper lobe. Schematic diagram of the ROI. Single-energy and iodine-based images showed the pGGN.

Ground-glass nodule (GGN) refers to the pulmonary nodules with cloud-like increased focal density but lower than the internal vascular and bronchial shadows [2]. The lesions were divided into pure ground-glass nodule (pGGN) and mixed ground-glass nodule (mGGN) based on the foci components (Figures 1 and 2). Quite a few GGNs were confirmed as early lung adenocarcinoma or precancerous lesions. In this study, all the 76 GGN cases were pathologically confirmed early lung adenocarcinomas or precancerous lesions.

Since lung GGNs are histologically special, with small lesions and great affection by respiration, studies on GGN imaging at home and abroad are mainly focused on the characteristics of lesions in unenhanced CT scanning and follow-up according to GGN growth to confirm the doubling time [3–6]. There have been relatively few studies in China on the dynamic enhanced CT of early lung adenocarcinoma with GGNs. Chen et al. performed multislice spiral CT dynamic contrast-enhanced scanning in GGNs of early lung cancer. In their study, the CT net enhanced value, enhanced peak value, and peak time of ground-glass and solid components were measured. They found a longer peak time of ground-glass components than solid components with delayed enhancement. Chen et al. conducted the perfusion scanning on 24 cases of GGNs. Results of the perfusion curves suggested that the blood supply of early lung adenocarcinoma of the GGN did not reach the richness of solid nodular lung cancer, resulting in increased interstitial space and slowly rising branch of the enhancement curve. The overlapping perfusion curves of different pathological types of GGNs limited the differential diagnosis. Moreover, patients for CT perfusion received a high dose of X-ray, which made some injury to the human body. The regular CT obtains images of mixed energy, whose CT value is vulnerable to the beam-hardening effect, resulting in the “drifting” of the CT value; that is, the CT value on the image is inconsistent with the theoretical projection data. Therefore, the accuracy of CT values of the GGN is relatively low with regular CT.

As a new imaging technique, GSI can realize two sets of data sampling using instantaneous dual voltage (high and low bulb voltage of 80 and 140 kVp) in 0.5 ms. The attenuation coefficient of the X-ray can be measured by high-speed switching between the high and low energy. According to the known mass absorption coefficient of different substances changed with energy, the absorption of the X-ray in each energy point is calculated, and the single-energy imaging can be realized. The separation of components of tissues helps produce two groups of pairwise base matter density map. The quantitative measurement of the concentrations of base matter provides quantitative information for the correct diagnosis of the lesion [3–6]. Iodine is the main component of the contrast agent of enhanced CT, whose concentrations are measured in GGN lesions after contrast enhancement of iodine-based images. Concentrations of iodine reflect the hemodynamics of tissues with high differentiation among components of tissues. Normalized iodine concentrations (NIC, IC of foci/IC of the descending aorta in the same layer) used in this study reduced the impact of individual differences on the study among patients with different GGNs of early lung adenocarcinoma.

In different phases of enhanced CT, pairwise comparisons among CT values of the solid and ground-glass components of the GGN suggested no statistical significance. Theoretically, the enhanced CT scan reflects the blood supply of the lesion and the diffusion rate of the contrast agent. Different pathological types of GGNs have different tissue structures, angiogenesis, and vascular distribution, and the degree of enhancement should be different in each phase of enhanced CT. However, the small foci of lung GGNs, the less richness of the blood supply of GGNs than solid nodules, vulnerability to artifacts of respiratory, cardiac, large vessels, and blood flow, volume effects, etc., might explain that pairwise comparisons of CT values in each enhancement phase had no statistical significance.

Characteristics of GGNs were measured with NIC in the spectral mode. Pairwise comparisons among NIC values of the solid and ground-glass components of the GGN suggested statistically significant differences among all the phases. This indicated that NIC can truly reflect the iodine distribution and the blood supply of the lesion. The pathological manifestations of early lung adenocarcinoma with the GGN are tumor cells growing the alveolar septa, thickening of alveolar walls, incomplete occlusion of the alveolar cavity, and a small amount of mucus or exfoliated creeping tumor cells [7]. Iodine-based images can sensitively identify contrast agents with iodine in lesions, providing accurate information on the enhancement and acutely reflecting the slight enhancement of local foci. Thus, the spectral CT imaging is meaningful for the GGN with relatively weak enhancement and small lesions.

There are some limitations in this study. Most GGN cases were followed up, with relatively long follow-up periods, resulting in limited cases to collect. Further studies with large sample size are warranted to explore the value of spectral CT in the differential diagnosis of different pathological types of GGNs.

## Figures and Tables

**Table 1:** CT values of solid and ground-glass components in unenhanced and different phases of enhanced scanning.

CT values (Hu)	Unenhanced scanning	Arterial phase 35 s	Venous phase 75 s	Delayed phase 120 s
Solid components	−127.56 ± 120.56	−87.66 ± 144.82	−78.18 ± 134.89	−95.94 ± 158.41
Ground-glass components	−433.96 ± 106.84	−396.39 ± 153.60	−381.24 ± 112.12	−385.19 ± 120.91

**Table 2:** NIC of solid and ground-glass components of the GGN in different phases of enhanced gemstone spectral CT scanning.

NIC	Arterial phase 35 s	Venous phase 75 s	Delayed phase 120 s
Solid components	0.33 ± 0.16	0.52 ± 0.25	0.58 ± 0.34
Ground-glass components	0.21 ± 0.13	0.42 ± 0.25	0.49 ± 0.39

**Table 3:** *P* values of pairwise comparisons on parameters of solid components in the GGN.

Groups	Arterial/venous phase	Venous/delayed phase	Arterial/delayed phase
CT values	0.284	0.205	0.338
NIC	0.010	0.006	<0.001

*P* < 0.05 was considered statistically significant.

**Table 4:** *P* values of pairwise comparisons on parameters of ground-glass components in the GGN.

Groups	Arterial/venous phase	Venous/delayed phase	Arterial/delayed phase
CT values	0.491	0.719	0.557
NIC	<0.001	0.005	0.001

*P* < 0.05 was considered statistically significant.

## Data Availability

The analyzed datasets generated during the study are available from the corresponding author upon reasonable request.

## References

[1] Xu Y., Zheng M., Wang N., Wang R. (2019). Comprehensive study of surgical treated lung adenocarcinoma with ground glass nodule component. *Medical Science Monitor*.

[2] Chen K.-N. (2020). The diagnosis and treatment of lung cancer presented as ground-glass nodule. *General Thoracic and Cardiovascular Surgery*.

[3] Jia Y., Xiao X., Sun Q., Jiang H. (2018). Gemstone spectral imaging in lung cancer. *Medicine (Baltimore)*.

[4] Yang F., Dong J., Wang X., Fu X., Zhang T. (2017). Non-small cell lung cancer: spectral computed tomography quantitative parameters for preoperative diagnosis of metastatic lymph nodes. *European Journal of Radiology*.

[5] Lin J.-Z., Zhang L., Zhang C.-Y., Yang L., Lou H.-N., Wang Z.-G. (2016). Application of gemstone spectral computed tomography imaging in the characterization of solitary pulmonary nodules. *Journal of Computer Assisted Tomography*.

[6] Wang Y.-W., Chen C.-J., Huang H.-C. (2021). Dual energy CT image prediction on primary tumor of lung cancer for nodal metastasis using deep learning. *Computerized Medical Imaging and Graphics*.

[7] Zhang Y., Fu F., Chen H. (2020). Management of ground-glass opacities in the lung cancer spectrum. *The Annals of Thoracic Surgery*.

